# Evaluation of a curved surgical prototype in a human larynx

**DOI:** 10.1007/s00405-021-06791-9

**Published:** 2021-04-22

**Authors:** L. R. Schild, F. Boehm, L. Kienle, A. Seitz, L. A. Kahrs, T. M. Boeckers, J. Greve, T. K. Hoffmann, P. J. Schuler

**Affiliations:** 1 Department of Otorhinolaryngology, Head and Neck Surgery, Ulm University Medical Centre, Frauensteige 12, 89075 Ulm, Germany; 2 Institute of Orthopaedic Research and Biomechanics, Centre for Trauma Research Ulm, Ulm University Medical Centre, Ulm, Germany; 3 Department of Mathematical and Computational Sciences, University of Toronto Mississauga, Mississauga, Canada; 4 Institute of Anatomy, Ulm University Medical Centre, Ulm, Germany; 5 Surgical Oncology Ulm, i2SOUL Consortium, Ulm, Germany

**Keywords:** Flexible instruments, Curved, Video laryngoscope, Laryngeal surgery, TORS

## Abstract

**Purpose:**

It is not always possible to create linear access to the larynx using a rigid operating laryngoscope for microlaryngoscopy. In this study, we evaluate the usability of a novel curved surgical prototype with flexible instruments for the larynx (sMAC) in a simulation dummy and human body donor.

**Methods:**

In a user study (*n* = 6), head and neck surgeons as well as medical students tested the system for visualization quality and accessibility of laryngeal landmarks on an intubation dummy and human cadaver. A biopsy of the epiglottis was taken from the body donor. Photographic and time documentation was carried out.

**Results:**

The sMAC system demonstrated general feasibility for laryngeal surgery. Unlike conventional microlaryngoscopy, all landmarks could be visualized and manipulated in both setups. Biopsy removal was possible. Visibility of the surgical field remained largely unobstructed even with an endotracheal tube in place. Overall handling of the sMAC prototype was satisfactorily feasible at all times.

**Conclusion:**

The sMAC system could offer an alternative for patients, where microlaryngoscopy is not applicable. A clinical trial has to clarify if the system benefits in clinical routine.

## Introduction

Microlaryngoscopic techniques such as transoral laser microsurgery (TLM) have become the gold standard for the treatment of early-staged laryngeal cancer. This approach offers most patients good oncological results, functional outcome and few complications [1]. Nevertheless, there are cases where the use of TLM is unsatisfactory or even impossible due to inadequate laryngeal exposure. Owing to the rigid and straight form of the standard operating laryngoscope, the cervical spine must be brought into a hyperextended position to allow for direct access to the larynx. Especially in patients with an immobilized cervical spine, insufficient mouth-opening, macroglossia, or scarring after previous radiotherapy, this is not practicable [2]. For these patients, therapeutic alternatives must be sought. In recent years, attempts have been made to identify patients with difficult access path to the larynx preoperatively using a predictive scoring system. The Laryngoscore, which was introduced in 2014 by Piazza et al., is based on certain preoperative clinical predictors such as thyromental distance and upper jaw dental status [3, 4]. Although this method may enhance the identification of suitable candidates for operative microlaryngoscopy, it has not yet become widely accepted. Therefore, the limited feasibility of TLM is still often only noticed intraoperatively, with difficult laryngeal exposure in about 15% of the patients [3]. As an alternative therapy option, transoral robotic surgery (TORS) can be considered, which has already been successfully applied in cases of impossible TLM [5]. Currently two systems are on the market that are clinically approved for robot-assisted laryngeal treatment: the DaVinci system (Intuitive Surgical, Sunnyvale, USA) and the Flex system (Medrobotics, Raynham, USA). The latest DaVinci single port SP system allows the transoral insertion of the endoscope as well as up to three fully wristed instruments through a 2.5 cm cannula. The instrument tips are triangulating, enabling a narrow working space. Advantages of the DaVinci SP as compared to the previous DaVinci Xi and Si in visualization and surgical precision were shown in an initial study [6]. However, there are anatomical, functional, and oncological limitations and contraindications for the use of the DaVinci System, with its mostly rigid instruments [7, 8]. Not least because here too, as with TLM, the patient's cervical spine must be brought into a hyperextended position. A more disruptive approach is taken by Medrobotics Flex System, which is highly versatile and able to reach, visualize, and manipulate laryngeal structures through its snake-like shape and flexible instruments. Although visualization and access to oropharyngeal structures, such as palatine tonsil area, posterior pharyngeal wall, and epiglottis are satisfying with the Flex system, it is highly challenging to manipulate the vocal folds [9].

Furthermore, in our hands, the technical and operational complexity of the system seems to be excessive for some procedures, including the excision of laryngeal tumor tissue. Both TORS systems have in common that they are cost-intensive in acquisition and maintenance. In addition, there is a lack of large-scale randomized studies between TORS and TLM, which could justify their application as a comprehensive first-line therapy in the head and neck area [7].

We have developed a new surgical prototype, the surgical MAC system (sMAC), which could enable laryngeal diagnostics and surgical treatment even in cases of difficult laryngeal exposure [10]. The sMAC system is composed of a hyper-angulated video laryngoscope modified with flexible instruments. Up to now, high feasibility of the system has been demonstrated when used on porcine larynx. Nevertheless, the system has not yet been evaluated on the human anatomy. Therefore, in this study we evaluated the feasibility of the sMAC system in a human simulation dummy and a body donor. We evaluated whether important laryngeal landmarks can be adequately visualized and manipulated—especially in the case of a difficult airway. The feasibility was demonstrated in a user study involving head and neck surgeons of the local Department of Otorhinolaryngology, Head, and Neck Surgery.

## Materials and methods

### Surgical system

**Fig. 1:**
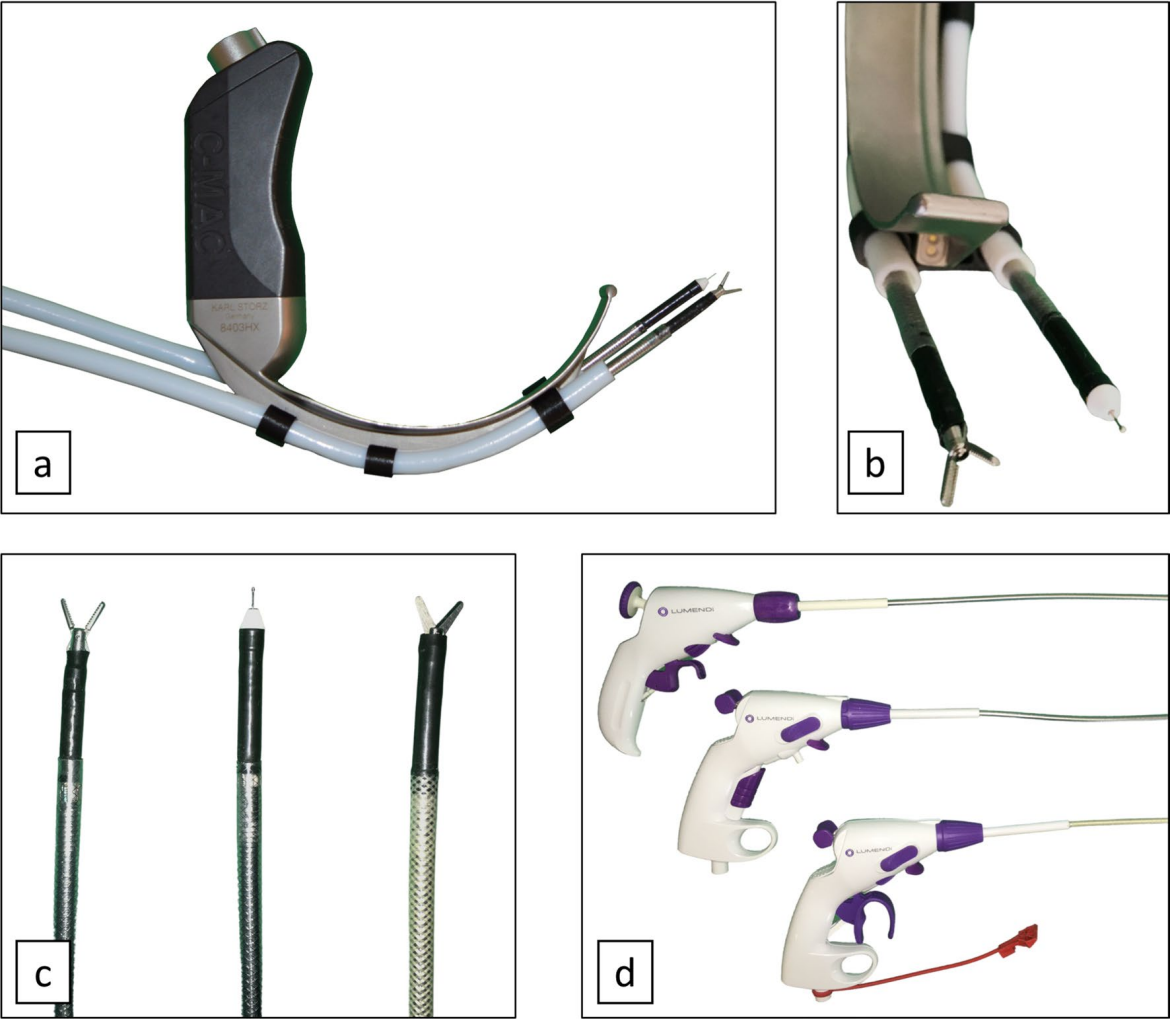
Curved prototype for transoral laryngeal surgery: the sMAC system. **a** The prototype is based on a hyperangulated video laryngoscope. Working channels made of low-friction polytetrafluoroethylene allow control over fully flexible and customized surgical instruments. **b** Light and video unit of the video laryngoscope. Exemplary assembly with gripping instrument and monopolar needle. **c**–**d** Fully flexible surgical instruments: grasper, monopolar needle, scissors

A detailed setup of the surgical prototype has been published before [10]. We modified a video laryngoscope for surgical purposes by equipping it with flexible dual-arm instrumentation (Fig. 1a, b). As a basis we used the hyper-angulated C-MAC D-Blade video laryngoscope with built-in light source and camera (640 × 480 pixels, Karl Storz, Tuttlingen, Germany). The shape of the blade was developed for intubation especially in a difficult airway. Rapid prototyped clips were inserted into the catheter guide of the C-MAC, attaching the 6 mm working channels on both sides. For these, we used Polytetrafluoroethylene (PTFE) as material to ensure frictionless sliding of the flexible instruments. The manually operated endoscopic tools DiLumen grasper, scissors, and monopolar needle with a diameter of 6 mm were used as instruments (Lumendi, Westport, USA) (Fig. 1c). Depending on the type of instrument, different steering mechanisms were implemented on the controllers (Fig. 1d). These surgical tools were originally developed for endoscopic colon surgery, but were shortened by the manufacturer to a length of 55 cm to meet our specific requirements in the head and neck area. The tools can be tilted up to 90 degrees in polar angle and 360 degrees in azimuthal angle, resulting in a spherical workspace. The instruments can be advanced up to 4 cm over the camera tip of the videolaryngoscope. The whole unit is attached to the operating table via a rapid prototyped bracket. A commercially available instrument holder is attached to the operating table and used to position the two instrument handles and fix them in place when not in use. For visualization the 7-inch standard monitor of the video laryngoscope was used (Karl Storz, Tuttlingen, Germany). In case of the human cadaver studies, the signal was transferred to a 40-inch flat screen TV (Sharp K.K., Osaka, Japan).

### Study structure

Experiments with the sMAC system were conducted on a conventional intubation dummy as well as on a human body donor.

### Intubation dummy setup

**Fig. 2:**
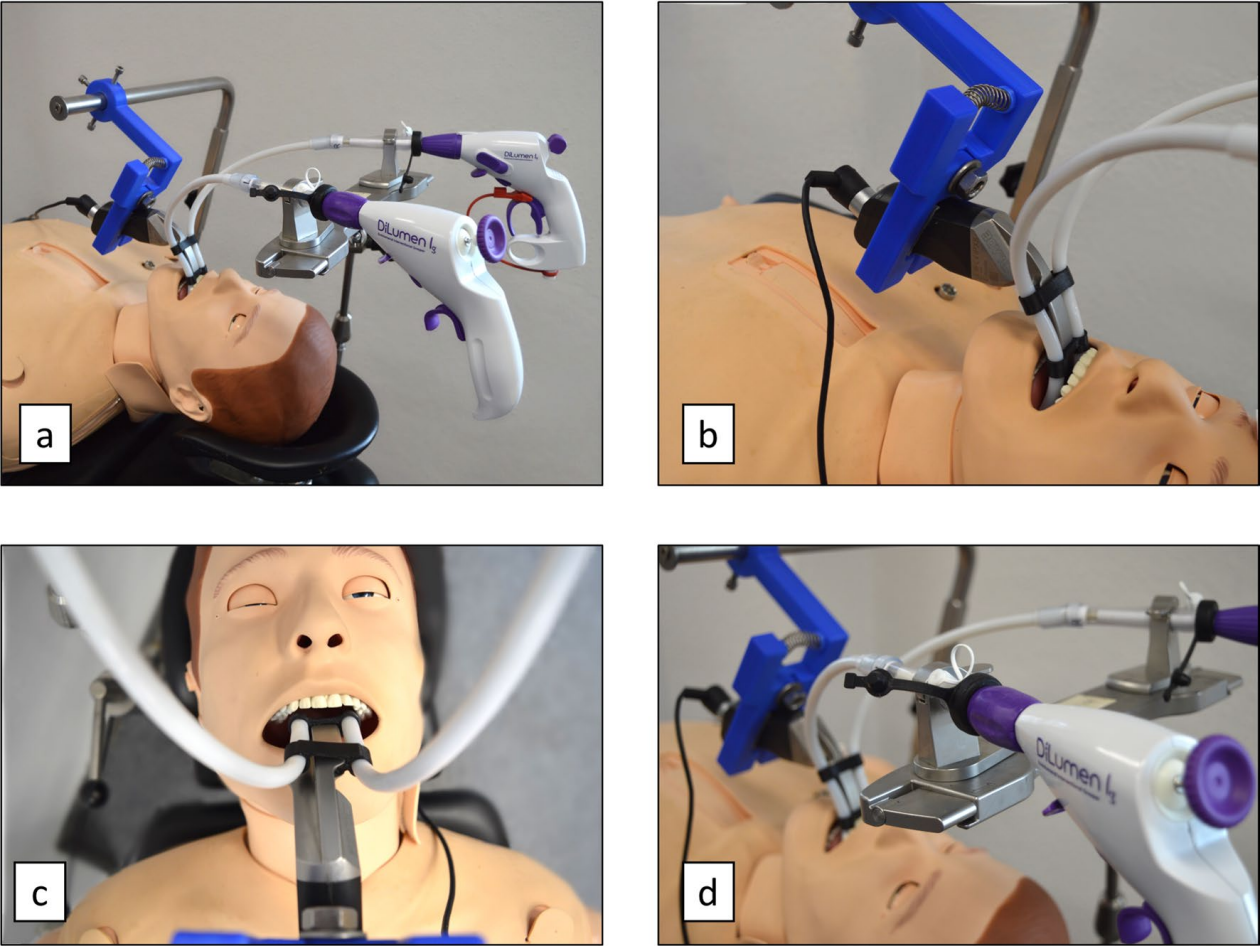
**a**–**d** Experimental setup of the intubation dummy study. In addition to the prototype, an instrument holder was fixed to the operating table, enabling bimanual manipulation. After indirect laryngoscopy and position control via a 7-inch monitor, the system was fixed with fully view on vocal fold level

For our experiments the standardized adult dummy Resusci Anne simulator (Laerdal, Stavanger, Norway) was placed on a surgical table. To simulate a surgical setting, a horizontal operating rod was attached to the table. The instrument holder was fixed in a position, which enabled the surgeon to work on the head end of the table. The sMAC system was inserted into the oral cavity by the surgeon in slight hyperextension of the cervical spine, performing an indirect laryngoscopy. Visualization and position control were provided using the 7-inch standard monitor (Karl Storz, Tuttlingen, Germany). Once the correct position was reached, the rapid prototyped bracket was attached to the shaft of the laryngoscope and fixed to the operating rod, locking the sMAC in position (Fig. 2).

### Intubation dummy user study

In a user study, medical students (*n* = 6) in their clinical stage of training tested accessibility and feasibility of the prototype according to a defined study protocol: After the grasper was introduced into the right working channel of the system, they were asked to visualize and access typical surgical landmarks such as left and right vocal folds, left and right false vocal folds, anterior commissure, postcricoid region, and ventral subglottis by means of the instrument tip. Each participant carried out this procedure twice in turn. Time was taken and photo documentation was performed.

In a second setup, an instrument exchange and the bimanual biopsy taking of the left vocal fold was simulated. For this purpose, a cutting surgical tool was inserted into the left working channel in addition to the grasper on the right hand side. It was the test persons' task to extract and reinsert the instruments to simulate an instrument change. Subsequently, the left vocal fold was grabbed with the grasper and manipulated using the cutting surgical tool. Time was taken and photo documentation was performed.

In a third setup, a tracheal tube was inserted under visual control to evaluate if, and to what extent, it may obstruct the visualization of important laryngeal structures, which was again documented by photographs.

### Cadaver setup

**Fig. 3:**
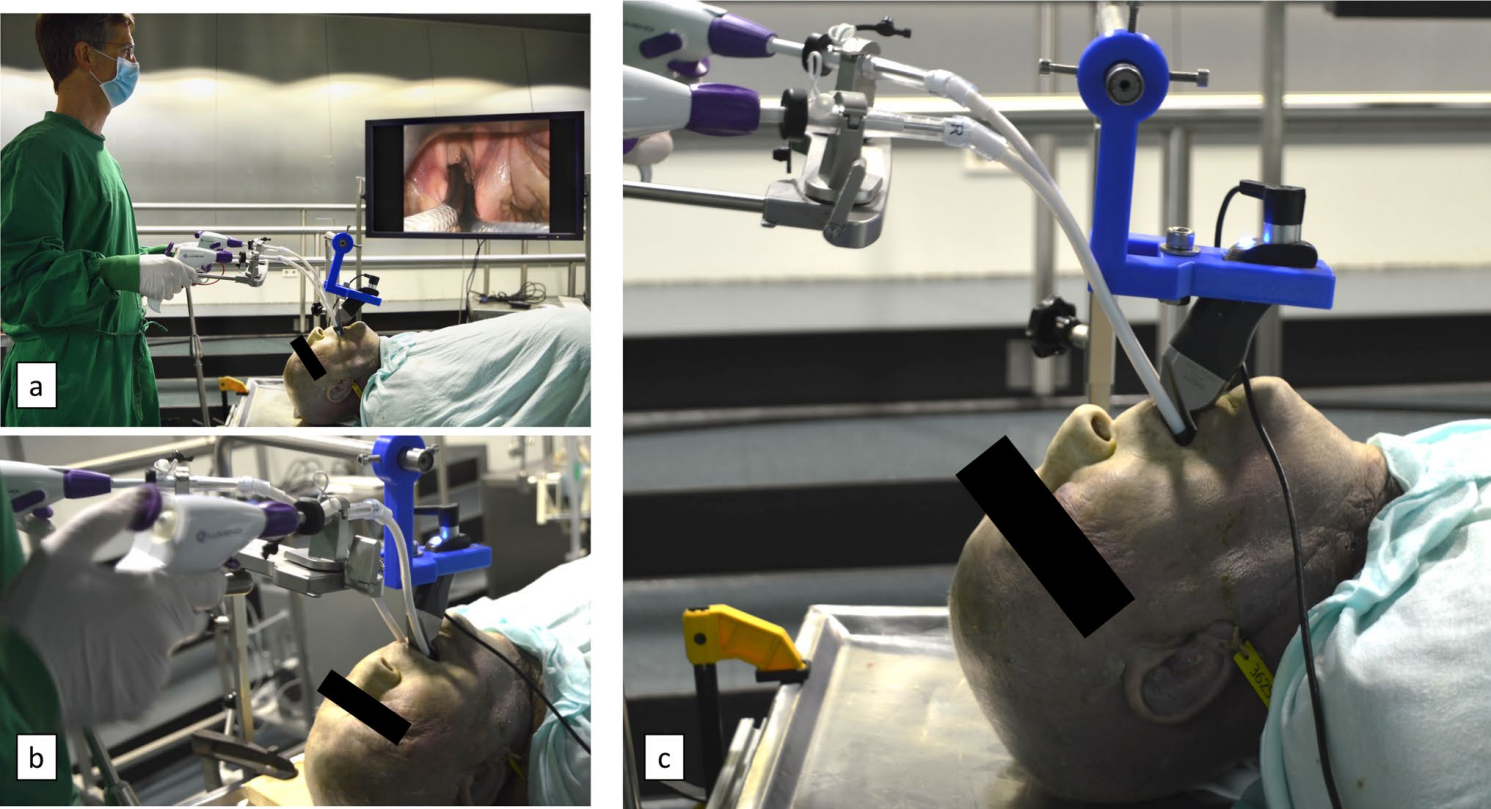
**a**–**c** Experimental setup of the body donor study. Body donor was placed on dissection table and the system attached. After suction, indirect laryngoscopy was performed and system fixed. Visualization was provided by 40-inch external monitor

The experiments involving human body donors were approved by the local ethics committee (# 89/19). We further evaluated the feasibility of the sMAC prototype for visualization and manipulation of the larynx on an adult human cadaver fixed in formalin in a simulated surgical setting. The physique of the male body donor was obese, and his formalin fixation allowed only limited hyperextension of the cervical spine and a mouth opening of about 2 cm, which suggested a difficult enoral approach to the larynx. The cadaver was placed on the operating table, while the operating rod and instrument holder were attached to it, similarly to the setup of the simulation dummy experiments. The prototype was inserted into the oral cavity and the vocal fold level was adjusted (Fig. 3). Visualization was provided through a 40-inch flat screen TV. Once the system was correctly positioned, it was fixed by its clamp to the surgical crossbar. The flexible surgical tools were introduced by the surgeon. We then conducted the user study with the sMAC on the cadaver, as well as biopsy collection of the epiglottis tip, done by an experienced head and neck surgeon.

### Operating laryngoscope

On the human cadaver we further compared visualization between a conventional microlaryngoscope and the sMAC system in this case of difficult airway. For microlaryngoscopy we used the Kleinsasser operating laryngoscope OP 292 (Aesculap, Tuttlingen, Germany) with its chest support. Visualization was enabled using a smartphone camera with an attached endoscope to smartphone adapter (SMART SCOPE, Karl Storz, Tuttlingen, Germany) and rigid endoscope (0°).

### Cadaver user study

Prior to the application of the sMAC system, vocal fold level should be visualized by a conventional operating laryngoscope to later be able to compare the visualization quality between the systems. In this user study, the participants with different levels of experience in transoral surgery (three senior physicians, one resident, two medical students, *n* = 6) of the local Department of Otorhinolaryngology, Head, and Neck Surgery were asked to visualize and manipulate selected anatomical landmarks of the larynx by means of a right-handed instrument. Thus, as with the simulation dummy, anterior commissure, both vocal and false vocal folds, postcricoid region, and subglottis should be reached with the grasper. Afterwards, every participant carried out an exchange of the grasper instrument. The respective times for reaching the landmarks and changing the instrument were documented.

### Biopsy collection of the epiglottis

A small excision of the epiglottis tip should be performed, and a biopsy taken. To do so, the surgical prototype was aligned and fixed at a slightly higher than vocal fold level to fully expose the epiglottis. As instrumentation, the grasper was inserted in the right, the mechanical scissors in the left working channel.

## Results

### Handling of the sMAC

In the present study, the sMAC system showed easy and quick attachment at different required heights and angles on the dummy and the cadaver. Glottic and supraglottic landmarks could be exposed in any case and the sMAC could be intraoperatively locked in position by the rapid prototyped bracket. The integrated endoscopic camera provided stable visualization of oropharyngeal landmarks and enabled the surgeon to follow their instrument movements on the 7-inch standard monitor, as well as a 40-inch external display at eye level.

After a short introduction to the different instrument controls of the surgical instruments—grasper, scissors, and monopolar needle—the handling in a preliminary exercise was practicable for all participants. Although grasper and monopolar needle could be tilted up to 90° (polar angle) in every spatial direction via joystick-like control by thumb, resulting in a spherical working area, the cutting tool only allowed movement in the single-dimensional space. Any other movements had to be made possible by rotating the entire controller, which complicated the navigation. Inside the oropharynx bimanual control of the instruments was continuously feasible and thus enabled reaching and grasping of various anatomical structures of the larynx at different levels of depth.

The video image as well as the mechanically transmitted haptic impressions of the instruments enabled satisfactory depth perception. Exchange of the surgical instruments could be carried out by each subject. The intensity of illumination of the video laryngoscopes LED was sufficient for surgical purposes. Especially coarser structures such as anterior commissure or arytenoid cartilage could be displayed in sufficient quality both on the 7-inch standard monitor and the 40-inch external monitor. However, the resolution of the integrated camera in the C-MAC video laryngoscope is not sufficient for the identification of structural details.

### Intubation dummy user study

**Fig. 4:**
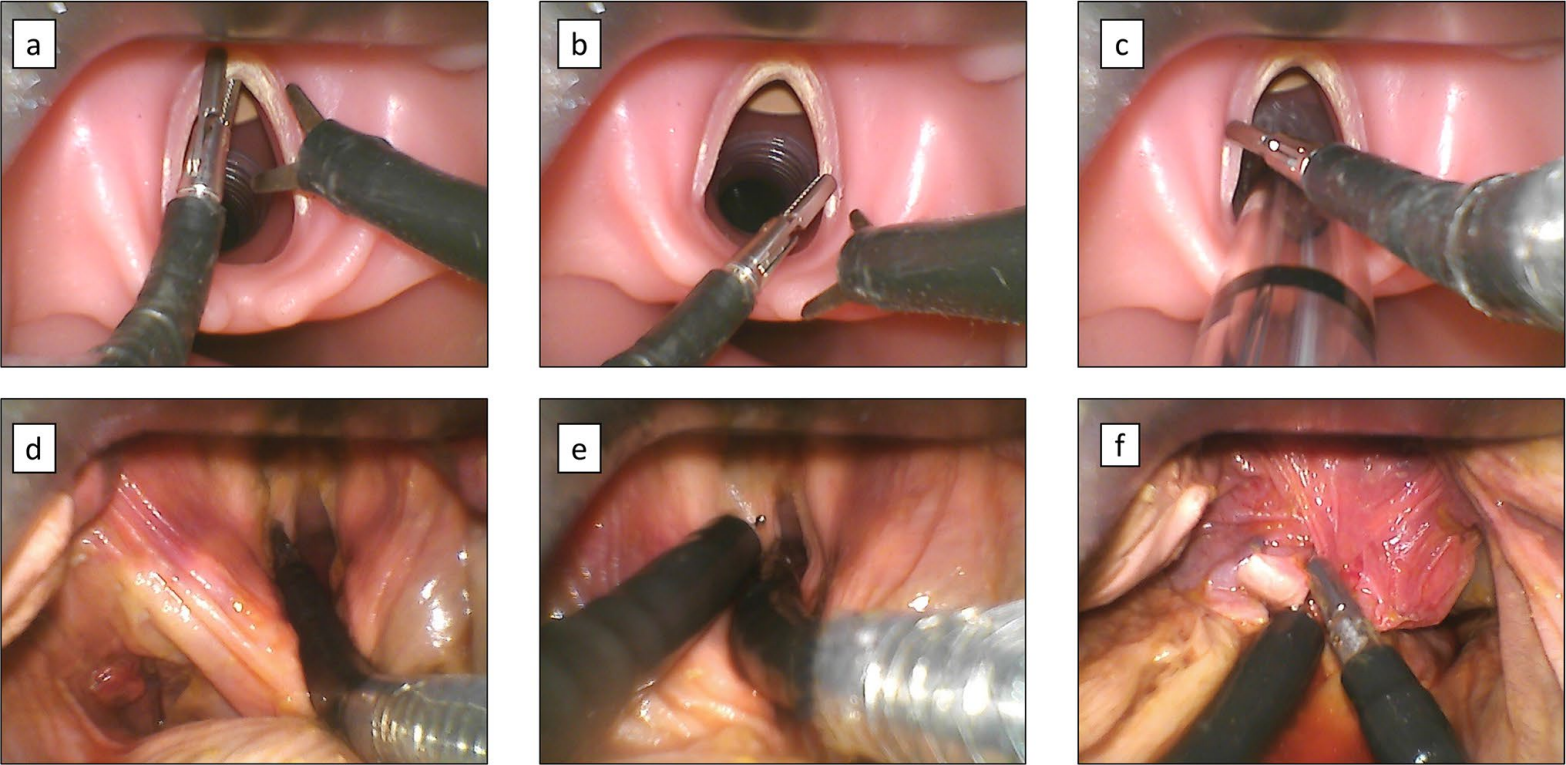
Intubation dummy (**a**–**c)** and body donor (**d**–**f**) user study: photographic documentation with the sMAC system. **a** Excision position: Grasping of the anterior commissure and holding the cutting instrument ready. **b** Grasping of the right vocal fold. **c** Manipulation of the left vocal fold possible despite intubation. **d** Visualization and reachability of left vocal fold. **e** Bimanual manipulation using grasper and monopolar needle. **f** Biopsy taking of the distal tip of the epiglottis

Visualization, accessibility, and manipulation of the dummy’s left and right (false) vocal folds, anterior commissure, postcricoid region, and ventral subglottis was always achieved by the participants (Fig. 4a–c). The time needed was 90.7 ± 25.8 s in the first, respectively, 49.0 ± 7.6 s in the second attempt. Furthermore, it was possible to perform the dual instrument exchange and to bring the surgical instruments into a position suitable for vocal fold excision for an average of 127.8 ± 32.3 s (Fig. 4a). It was possible to intubate the simulation dummy by means of an endotracheal tube under sight. In this case the visualization of the anterior commissure was still possible without major obstacles, as shown in Fig. 4c.

### Cadaver study

The formalin fixation of the body donor resulted in a stiffened cervical spine and a limited mouth opening of approximately 2 cm. It was, therefore, impossible to examine the supraglottic structures or the vocal fold level using the conventional Kleinsasser operating laryngoscope even after multiple forceful attempts. On the other hand, the subjects were able to conduct an indirect laryngoscopy, while follow the endoscopic image on an external monitor. It was always possible to visualize vocal fold level using the hyper-angulated blade of the sMAC system. In the cadaver user study, 6/6 participants were able to reach and manipulate important laryngeal landmarks using the sMAC: bilateral vocal folds and vestibular folds, anterior commissure, postcricoid region as well as subglottis. The participating surgeons required an average of 33.0 ± 7.0 s. In addition, exchanging the grasper tool was always possible without complications in a time period of 23.1 ± 4.3 s. Photo documentation of exemplary manipulations are shown in Fig. 4d–f. The relatively great distance of the camera unit to the vocal folds, and thus the lacking magnification on the external monitor was not satisfactory, because the sMAC system could not be inserted deeper into the oropharyngeal corridor due to the stiffened cervical spine of the body donor.

### Biopsy collection of the epiglottis

A biopsy from the specimens’ epiglottis tip could be successfully excised in three cuts and extracted, while tissue control could be maintained at any times (Fig. 4f). The epiglottis was grasped at the most cranial end, pulled in tongue base direction and dissected using the mechanical scissors. The approximately 1 cm × 1 cm biopsy specimen were then removed transoral. Owing to the flexibility of the tissue, this was possible despite the fact that the diameter of the working channel was only 8 mm.

## Discussion

The usability of the sMAC system for the surgical manipulation of important laryngeal landmarks in a simulation dummy and adult body donor was demonstrated in this study. The structures could be visualized even in a difficult airway where conventional micro-laryngoscopy would not be possible. Handling of the sMAC prototype and its surgical instruments was possible at any time. Furthermore, rapid learning success was observed in the participants, as shown by the significant improvement in speed between the first and second round of landmark manipulation in the intubation dummy. The system could be attached quickly even in cases of varying requirements on angles and heights of the attachment. In this study, the sMAC system provided a free view of the vocal folds even in the intubated manikin, allowing manipulation of relevant laryngeal landmarks as well as exemplary excisions.

In the treatment of early staged (T1–T2) (supra)glottic carcinoma radio-chemotherapy and surgical treatment, especially TLM, are considered as standard treatments, which offer similar (good) results in oncological outcome [11]. In one of the few directly comparative retrospective databases analysis studies, Hanna et al. showed that also transoral robotic surgery (TORS) can achieve competitive oncological results, since there was no observed difference in margin status and necessitating adjuvant radiation compared to TLM [12].

Nevertheless, the difficulty in treating glottic lesions in cases with aggravated accessibility is a major problem for both TLM and conventional TORS. Approximately, 20% of the cases are intraoperatively considered to be partially or completely unsuitable for transoral surgery due to intensive trismus, altered cervical spine anatomy or scarring after previous radiation [8]. Both, TLM and TORS, have a need for straight line access in order to forward the endoscope, the laser beam or the rigid instruments to the larynx. This can only be achieved by a protruding hyperextension of the patient's cervical spine, which is for the above reasons not possible in every case. Inadequate visualization—especially of the anterior commissure—and considerable force on laryngeal structures and maxillary incisors can be associated with edema, mucosal bleeding, or tooth damage [13].

With regard to TORS, there are further aspects which could make the sMAC system a persuading alternative: unlike TORS the lack of haptics could be improved through continuous mechanical instruments and the waiver of physical separation between control console and intervention unit. In addition, TORS leads up to 90% higher costs as compared to established methods, while there is a lack of large-scale randomized trials that would show a clinical benefit for the patients [14].

Based on our evaluation, the additional costs for a surgical procedure assisted by a DaVinci system are approximately 6000 EUR (7100 USD). These costs are made up of maintenance charges, acquisition costs, as well as instrument acquisition and reprocessing [15]. Likewise, the cost of a procedure with the s-MAC system consists of investment and operating costs: The C-MAC D-BLADE videolaryngoscope and suitable monitor, which are already available in most hospitals, can be seen as initial investments. The remaining components of the s-MAC system are designed as disposable products, thus providing a hygienic and cost-effective option. At the current stage, the total costs for the 3D-printed bracket, clips, and working channels are therefore around 10 EUR per procedure. The flexible instruments used are prototypes that Lumendi has developed and produced in a small series especially for this project; the actual costs for these cannot yet be realistically estimated at a fixed amount. The instrument holder was also designed by Lumendi and adapted to the instruments. However, we assume that in the case of a larger series at a later date, prices per piece will be within the usual market prices for single-use endoscopic instruments. Roughly speaking, the s-MAC system can be expected to have an investment cost of less than 20,000 EUR, with disposable products bringing the price per procedure down to a fraction of the same for RAS. However, it should be noted that prices may increase upon approval of the system as a medical device due to the high-quality requirements placed on the material.

The hyper-angulated form of the sMAC system offers a more natural approach which is better adapted to the anatomical conditions of the oropharyngeal space than previous established surgical systems. Therefore, it could be shown in previous experiments, that the sMAC system reduces the force applied to maxillary incisors and supraglottis significantly in contrast to a conventional operating laryngoscope. In the scenario of a stiffened cervical spine, the system could demonstrate a 40% peak force reduction acting on the maxillary incisors and a 65% reduction of the average intraoperative force on the supraglottis [16].

In the body donor setup of this study, the sMAC system demonstrated adequate visualization for surgical purposes even under challenging conditions, where conventional microlaryngoscopy was not feasible. In a clinical context, up to now such a patient would only have the surgical option of an open-neck approach, such as a supraglottic laryngectomy. However, as compared to microlaryngoscopic techniques as TLM, this open approach may result in significant worse functional outcomes, such as swallowing as well as longer hospitalization time, feeding tube duration, and tracheotomy duration as compared to TLM [17]. It is also of interest when comparing the functional and oncological results of open supraglottic laryngectomy and transoral robotic supraglottic laryngectomy. While there are no differences in overall survival time and disease-specific survival time between groups, the open access operated cohort had a longer oral feeding time, hospitalization and recovery period [18]. With this knowledge, the goal must be to enable noninvasive surgery for patients who are not treatable by TLM and conventional TORS, such as with the DaVinci System. The novel sMAC could become such an alternative treatment option.

However, due to the stiffened cervical spine of the body donor, the system could not be inserted as deep into the oropharynx as desirable, resulting in a camera image which is adequate for the visualization of supraglottic, but not glottic structures such as the vocal folds. The possibility of a closer imaging of petite structures would highly improve the quality and safety of the procedure. A (digital) zoom function would be conceivable and desirable here, while requiring a higher resolution of the camera chip. In terms of visualization, the sMAC system allows the exposure of important laryngeal landmarks, but its quality is not competitive with established systems. In contrast, the modern DaVinci models use two HD endoscope cameras for visualization, resulting in a 3D image for the surgeon. There is emerging evidence that such 3D systems provide benefit for the surgical performance [19]. In addition to a high video resolution, systems like the DaVinci SP further have a zoom function, which enables adequate magnification of smaller laryngeal structures for the surgeon. In transoral laser micro-laryngoscopy, an operating microscope enables a high-quality visualization and zoom. The video laryngoscope used in our system was not originally designed for surgical purposes, but rather to locate the glottis and place a tube under visual control. Accordingly, no high-definition camera was used, but only a 640 × 480 pixel camera. Thus (I), the quality of the camera image and (II) the possibility of a (digital) zoom are two elementary points that should be improved in the sMAC system in the future in order to be considered serious competition to established procedures in the clinic.

In summary, the limitations of the system are the insufficient camera quality and the lack of approval. Limitations of the pilot study are the low number of body donors and the limited quantification of the findings.

## Conclusion

The sMAC system is a promising approach in laryngeal surgery. Its major advantage is its potential for use in difficult anatomical conditions where a straight trajectory is not suitable. Challenging landmarks, such as the anterior commissure, can be visualized and accessed, the biopsy collection can be performed. Compared with conventional TORS systems, the system offers significantly reduced costs, easier handling, and intraoperative haptic feedback. The visualization quality of laryngeal fine structures is not competitive as compared to TORS and TLM and needs to be optimized. Taking into consideration the prototype stage of the system, the small subject collective and the limited number of body donors, further investigations and first clinical studies are necessary to identify added value, weaknesses, and indications of the sMAC system.

## Data Availability

Data available on request from the authors.
